# Whole genome sequencing of three cloacal bacterial isolates from migratory birds

**DOI:** 10.1128/mra.00216-26

**Published:** 2026-05-29

**Authors:** Girish Kumar, Susan S. Pagano, Nicole Cavanaugh, André O. Hudson, Michael A. Savka

**Affiliations:** 1 The Thomas H. Gosnell School of Life Sciences, Rochester Institute of Technology 6925, Rochester, New York, USA; University of Maryland School of Medicine, Baltimore, Maryland, USA

**Keywords:** migratory passerine, cloaca, pathogen, microbiome, biogeography

## Abstract

We report the whole-genome sequences of three bacterial isolates from the cloaca of migratory songbirds. Among these, *Kluyvera cryocrescens* and *Stenotrophomonas rhizophila* are known as opportunistic human pathogens. In contrast, *Pseudomonas mandelii* is a psychrotrophic bacterium that is generally considered non-pathogenic.

## ANNOUNCEMENT

Migratory birds serve as critical bio-vectors, facilitating the long-distance dissemination of diverse microbial communities across geographical landscapes (1). The avian cloaca harbors a complex and dynamic microbiota that plays a significant role in host health and can serve as a reservoir for environmental and potentially zoonotic microorganisms (2). While extensive research has characterized the transient and luminal microbial populations within avian gastrointestinal tracts, the investigation into bacteria exhibiting a lifestyle associated with the cloacal environment remains underexplored. Understanding these bacterial communities is crucial, as their migratory hosts enable wide dispersal, influencing wildlife health, ecosystem balance, and potential pathogen transmission to animals and humans.

The samples were collected in Fall 2017 from migratory passerines—Gray Catbird (*Dumetella carolinensis*) and White-throated Sparrow (*Zonotrichia albicollis*)—at Braddock Bay Bird Observatory, Hilton, NY (43.323367, −77.71736). Each bird was sampled for culturable bacteria by swabbing the cloacal mucosa with a sterile cotton swab. Swab tips were placed in sterile vials with 50% glycerol/50% tryptic soy broth (TS), vortexed, and stored at −20°C. The cloacal swab was then inoculated into TS medium and incubated at 28°C for 3 days. The resulting culture was serially diluted (10-fold) and plated on TS agar media and incubated under the same conditions to isolate colonies. From the multiple colonies observed on the medium, three distinct colonies exhibiting different morphologies were selected and subcultured twice on TS agar medium at 28°C for 48 hours to ensure purity. Bacterial isolates RIT-550 and RIT-551 were cultured from the catbird, while RIT-562 was cultured from the sparrow cloacal sample (Table 1). The field sampling procedures of this study were approved by the RIT Institute Animal Care and Use Committee (RIT IACUC proposal 2011-06) (rev 2017).

**TABLE 1:** Genome annotation information for the three bacteria associated with the cloaca of two species of migratory birds

Sample	# Raw reads	# Bases (million)	SRA accession	Assembly accession	Best species hit (accession)	ANI^*a*^ (%)	Assembly size (bp)	Coverage (X)	# Contigs	N50	GC (%)	# Genes
RIT-550	965157	454.1	SRR22247048	GCA_026241935.1	*Kluyvera cryocrescens* (GCA_001571285.1)	99.33	4,718,047	90.46	28	339,294	54.9	4,440
RIT-551	1060551	495.3	SRR22247047	GCA_026241915.1	*Stenotrophomonas rhizophila* (GCA_000661955.1)	87.45	3,972,609	83.88	32	254,784	66.6	3,571
RIT-562	802184	374.7	SRR22247046	GCA_026241895.1	*Pseudomonas mandelii* (GCA_007858265.1)	90.94	6,347,377	52.99	102	183,867	59.5	5,788

^*a*^ ANI, average nucleotide identity.

To extract genomic DNA (gDNA) from each of the three isolates, we used 25 mg of bacterial culture grown in TS broth for 24 hours at 28°C with shaking, employing the E.Z.N.A. bacterial DNA kit (Omega Bio-Tek, Norcross, GA). For the library preparation, 1 ng of gDNA was utilized from each sample following the standard procedure outlined in the Nextera XT library preparation kit (Illumina, San Diego, CA). The libraries were then quantified using a Qubit 3.0 fluorometer and diluted to 16 pM prior to sequencing on an Illumina MiSeq (v3 chemistry, 2 × 300 cycles).

Default parameters were used for all software unless specified. After quality control processing with the program fastp (3), the paired-end raw reads were *de novo* assembled into contigs using the SPAdes genome assembler ([4]; version 3.5.0). Species identification was carried out with GTDB-Tk version 2.5.2 (5) by analyzing average nucleotide identity (ANI) and alignment fraction. The annotation of sequences was performed by utilizing the NCBI Prokaryotic Genome Annotation Pipeline (6). Among the three genomes that were sequenced, two (*Kluyvera cryocrescens* and *Stenotrophomonas rhizophila*) were previously identified as opportunistic human pathogens (7, 8). In contrast, *Pseudomonas mandelii* is a non-pathogenic, environmental bacterium found in natural mineral water, soil, and agricultural fields (9). This work enhances our understanding of the avian cloacal microbiome, its ecological and epidemiological roles, and migratory birds’ influence on microbial biogeography.

## Data Availability

The whole-genome assemblies and Sequence Read Archive (SRA) accession numbers of the bacterial genomes are presented in Table 1, and can be downloaded from GenBank and SRA, respectively.
